# TRIM50 Inhibits Proliferation and Metastasis of Gastric Cancer via Promoting *β*-Catenin Degradation

**DOI:** 10.1155/2022/5936753

**Published:** 2022-08-22

**Authors:** Rongzhou Li, Peng Xu, Xiaosheng Jin, Zhengchao Shi, Qingqing Zhang, Fangpeng Ye, Weilai Yu, Tingting Ji

**Affiliations:** ^1^Department of Gastroenterology, Ruian People's Hospital, Ruian City 325200, Zhejiang Province, China; ^2^Third Affiliated Hospital of Zhejiang Chinese Medical University, Hangzhou City 310005, Zhejiang Province, China

## Abstract

**Background:**

Gastric cancer (GC) is a common malignancy with a poor prognosis. Tripartite motif-containing 50 (TRIM50) belongs to the TRIM family and is reported to be related to numerous cancers. This study aimed to investigate the function of TRIM50 in GC.

**Methods:**

Three microarray datasets (GSE13911, GSE79973, and GSE19826) containing GC and adjacent nontumor tissues were used for bioinformatics analysis to screen GC-related genes and assess the associations between GC development and TRIM50 expression. Then, TRIM50 expression in GC cells was detected at mRNA and protein levels. After TRIM50 was knockdown or overexpressed, the effect of TRIM50 on the proliferation and metastasis of GC cells was analyzed using Cell Counting Kit-8 (CCK-8), flow cytometry, scratch, and Transwell assays. The interaction between TRIM50 and *β*-catenin was analyzed. The expression of cell cycle-, migration-, invasion-, and Wnt/*β*-catenin signaling pathway-related proteins was detected by Western blot. Furthermore, we measured the role of TRIM50 overexpression on tumor growth as well as the Wnt/*β*-catenin signaling pathway *in vivo*. In addition, XAV939 (a WNT/*β*-catenin signaling pathway inhibitor) was used to clarify the mechanism of TRIM50 on GC.

**Results:**

Bioinformatics revealed that TRIM50 expression was decreased in GC samples and associated with GC development. *In vitro* study revealed that TRIM50 overexpression impeded the GC cell proliferation and metastasis, while TRIM50 knockdown presented the opposite results. In addition, TRIM50 interacted with *β*-catenin to induce the degradation of *β*-catenin. In *in vivo* assay, TRIM50 overexpression inhibited tumor growth and blocked the Wnt/*β*-catenin signaling pathway. In addition, TRIM50 knockdown-promoted cell proliferation and metastasis in GC cells were inverted by XAV939.

**Conclusion:**

TRIM50 overexpression may inhibit cell proliferation and metastasis in GC via *β*-catenin degradation, indicating that TRIM50 could be a target for the treatment of GC.

## 1. Introduction

Gastric cancer (GC) is one of the most common malignancies of the digestive tract worldwide [1, 2]. Based on the GLOBOCAN's 2020 database statistics, the incidence and mortality of GC are 7.1% and 9.1%, respectively [3]. The incidence of GC differs as per race, gender, or lifestyle. In some area, GC even has a higher incidence than lung cancer, which is considered the most common cancer globally [4, 5]. Though the incidence of GC in many countries is declining, it remains a major cause of cancer-associated mortality in China [6, 7]. Owing to the absence of significant symptoms in early GC, most GC patients have already reached middle or advanced stages when diagnosed, which causes a dismal prognosis [8]. Hence, it is an urgent need to further investigate the mechanism of GC and find an efficient target for its treatment.

Recently, the study on protein degradation has become a focus in oncology [9]. As a vital mediator of the Wnt/*β*-catenin signaling pathway, *β*-catenin takes part in intercellular adhesion as well as modulation of cell activities [10]. Specifically, *β*-catenin binds to the nuclear transcription factors to form complexes that regulate the expression of the downstream proteins (c-Myc and Cyclin D1) and induce malignant transformation in cells [11]. Some investigators have found that *β*-catenin degradation correlate with GC progression. For instance, Higashimori et al. have demonstrated that forkhead box F2 (FOXF2) can suppress GC via upregulating degradation and ubiquitylation of *β*-catenin [12]. In addition, Yang et al. have reported that F-box and WD-repeatdomain-containing 2 (FBXW2) can impede migration, invasion as well as metastasis of lung cancer cells by degrading *β*-catenin [13]. Thus, facilitating the degradation of *β*-catenin may be considered as a valid approach for GC treatment.

Tripartite motif-containing 50 (TRIM50) is a newly identified member of the TRIM family [14]. A published study conducted by Ma et al. has suggested that TRIM50 hampers the progression and development of hepatocellular carcinoma (HCC) *in vitro* by directly targeting Snail as well as reversing the process of epithelial-mesenchymal transition (EMT) [15]. Another research has demonstrated that TRIM50 is a tumor suppressor in ovarian cancer by reducing Src activity [16]. Up to now, numerous TRIMs, such as TRIM59 [17] and TRIM28 [18], have been demonstrated to be associated with the occurrence of GC. Nevertheless, the specific function of TRIM50 in GC has not been completely explored.

Hence, this study was designed to evaluate the effect and mechanism of TRIM50 in GC. We investigated TRIM50 expression in GC by bioinformatics and cell experiments, and then identified whether the cell proliferation, cycle progression, migration as well as invasion were modulated by TRIM50 in GC Cells. Furthermore, we investigated the specific mechanism of action of TRIM50 in GC. We found that TRIM50 expression was decreased in GC, and TRIM50 overexpression could prevent cell proliferation, cycle progression, migration as well as invasion of GC cells by promoting *β*-catenin degradation. This study will provide a novel scientific basis for the therapy of GC.

## 2. Materials and Methods

### 2.1. Microarray Data Collection and Bioinformatic Analysis

Three microarray datasets (GSE13911 (https://www.ncbi.nlm.nih.gov/geo/query/acc.cgi?acc=gse13911), GSE79973 (https://www.ncbi.nlm.nih.gov/geo/query/acc.cgi?acc=gse79973), and GSE19826 (https://www.ncbi.nlm.nih.gov/geo/query/acc.cgi?acc=gse19826)) were downloaded from National Center for Bioinformatics analysis (NCBI) Gene Expression Omnibus (GEO) database. The GSE13911 microarray included 69 GC patients, 31 adjacent nontumor tissues, and 38 GC tissues. GSE79973 and GSE19826 contained 10 adjacent nontumor tissues, 10 GC tissues, and 15 adjacent nontumor tissues, 12 GC tissues, respectively.

GEO2R, a convenient tool released by GEO database, was applied to obtain differentially expressed genes (DEGs). Adjusted *p*-values <0.05 and |log_2_FC| > 1 were considered as the criteria to select the DEGs. Then, the list of obviously down-regulated genes was exported separately. For identifying the overlapping DGEs in GSE13911, GSE79973 as well as GSE19826, a Venn diagram was created online (https://bioinformatics.psb.ugent.be/webtools/Venn/). Subsequently, Ualcan, a public portal that provides online analysis of The Cancer Genome Atlas (TCGA) data, was employed to analyze the relationship between TRIM50 relative expression and GC development.

### 2.2. Cell Culture and Transfection

Human gastric mucosal epithelial (GES-1) cells and human GC (AGS, BGC-823, HGC-27, MGC-803, MKN-28, MKN-45, SGC-7901) cells were supplied by Saibaikang Biotechnology Co., Ltd. (Shanghai, China). All cells were grown in RPMI-1640 medium containing 10% fetal bovine serum (FBS), except AGS which was cultured in F12 medium containing 10% FBS.

The short hairpin RNA (shRNA) that targeted human TRIM50 sequence was utilized for the transfection of MKN-45 and HGC-27 cells based on manufacturer's instructions. Cells transfected with shNC were served as the negative control. TRIM50 overexpression lentiviral vector was constructed through subcloning its coding sequence to the pLVX-Puro lentiviral vector. In short, 293 T cells were transfected with pLVX-Puro-TRIM50 using Lipofectamine 2000. After 48 h, recombinant lentiviral vectors were purified and utilized to infect SGC-7901 and AGS cells. Cells transfected with empty pLVX-Puro (vector) were used as the negative control. Quantitative PCR (qPCR) and Western blot were applied to measure the transfection efficiency.

### 2.3. Cell Counting Kit-8 (CCK-8) Assay

To determine the cellular proliferation ability, CCK-8 assay was carried out. Briefly, after being transfected with pLVX-Puro-TRIM50, shNC, or shTRIM50 for 48 h and treated with or without 10 *µ*mol/l of XAV939 (an inhibitor of the Wnt/*β*-catenin signaling pathway), the cells were collected and cultured in 96-well plates with 3 × 10^3^ cells/well. After 0, 24, 48, 72, and 96 h, CCK-8 reagent (HY-K0301, MCE, New Jersey, USA) was added to the cells and incubated for another 30 min. Subsequently, the light absorbance was measured in a microplate reader to evaluate cellular proliferation ability.

### 2.4. Flow Cytometric Analysis

The cell cycle was investigated using flow cytometric analysis. Upon being transfected with vector, pLVX-Puro-TRIM50, shNC, or shTRIM50 for 48 h and treated with or without 10 *µ*mol/l of XAV939, the cells were washed by phosphate buffer solution (PBS), fixed in 70% ice-cool ethanol, and then resuspended in DNA staining solution (containing PI and RNase A) and permeabilization solution. At last, the cell cycle phases were detected using a flow cytometer (C6, BD, San Jose, USA), and analyzed by the Flowing software.

### 2.5. Scratch Test

The migration ability of the cell was determined by a scratch test. Briefly, horizontal lines were evenly drawn on the back of 6-well plates with an interval of about 0.5–1 cm using a marker. Then, the cells transfected with vector, pLVX-Puro-TRIM50, shNC, or shTRIM50 for 48 h and treated with or without 10 *µ*mol/l of XAV939 were inoculated in the 6-well plates until covering the whole plate bottom. Subsequently, scratched was made on cells in the horizontal lines perpendicular to the back using a pipette tip. The floating cells were removed by PBS gently, and then the cells were cultured again in serum-free mediums. Following culturing for 24 h, the scratched wounds were viewed with a microscope, and analyzed by ImageJ.

### 2.6. Transwell Assay

The cell invasion ability was detected with the Transwell assay. Firstly, the upper Transwell chambers were precoated with 30 *μ*l diluted Matrigel at 4°C overnight. After that, the SGC-7901, AGS, and MKN-45 cells transfected with vector, pLVX-Puro-TRIM50, shNC, or shTRIM50 for 48 h and treated with or without 10 *µ*mol/l of XAV939 in serum-free medium were injected to the upper chambers. Dulbecco's modified Eagle's medium containing 10% FBS was placed to the lower chambers. After 24 h, the noninvaded cells in the upper chamber were completely erased using cotton-tipped swabs. The invaded cells on the lower chamber were fixed by formaldehyde (4%), stained with crystal violet (0.1%), and calculated under a microscope.

### 2.7. qPCR

The expression of TRIM50 mRNA was detected through qPCR. Total RNA from cells was isolated by Trizol (B511311, Sangon Biotech Co., Ltd., Shanghai, China). Subsequently, cDNA was synthesized with RNA reverse-transcription kit (CW2569, CWBIO, Beijing, China) based on the general protocols. Hereafter, qPCR was carried out with SYBR Premix Ex TaqII (RR820A, Takara, Dalian, China) to test the relative expression of TRIM50 mRNA. GAPDH was used as a housekeeping gene. The sequences of the qPCR primers used in this study are displayed in Table 1.

### 2.8. Western Blot

The protein levels were quantified by Western blot. In short, the total protein of the cells was extracted by radioimmunoprecipitation (RIPA) lysis buffer. Then, bicinchoninic acid (BCA) assay was applied to measure the protein concentration. Hereafter, the extracted protein was separated by 5% SDS-polyacrylamide gel electrophoresis (SDS-PAGE). After being transferred to polyvinylidene difluoride (PVDF) membranes and blocked in 5% skimmed milk, the protein was probed at 4°C with primary antibodies against TRIM50 (Ab272586), p21 (DF6423), p27 (AF6324), CDK4 (DF6102), cyclin D1 (Ab16663), Snail (AF6032), E-cadherin (AF0131), N-cadherin (AF4039), *β*-catenin (AF6266), c-Myc (AF0358), survivin (Ab76424), and GAPDH (AF7021) overnight. After washing 3 times, the membranes were reincubated with horseradish peroxidase (HRP)-conjugated second antibodies at room temperature for another 2 h. Signals were developed by enhanced chemiluminescent (ECL) reagents. The target protein was quantified using ImageJ. All primary antibodies were purchased from Affinity (Zhenjiang, China), except for antibodies against TRIM50, Cyclin D1, and survivin that were from Abcam (Cambridge, UK). All primary antibodies were used at a dilution of 1 : 1000, except for Cyclin D1 (1 : 100), Cyclin D1 (1 : 10000), and GAPDH (1 : 5000).

### 2.9. Co-immunoprecipitation (Co-IP) Assay

Co-IP assay was conducted to evaluate the interaction of TRIM50 and *β*-catenin protein. In brief, RIPA buffer was used to extract the total protein from AGS or SGC-7901 cells. Subsequently, the total protein was mixed with anti-TRIM50, anti-*β*-catenin, or normal IgG. Next, the samples were associated with protein A/G agarose beads. Followed by washing for 4 times, the samples were boiled in SDS-PAGE sample buffer for 5 min to elute the bead-bound protein. Finally, the proteins were analyzed by Western blot.

### 2.10. Protein Stability and Degradation Assays

Protein stability was assessed by using cycloheximide (CHX), a protein synthesis inhibitor. Briefly, after being transfected with vector or pLVX-Puro-TRIM50 for 48 h, the AGS and SGC-7901 cells were incubated with CHX (200 *μ*g/ml) for 3, 6, or 9 h. Subsequently, the cells were harvested and lysed, and *β*-catenin protein expression was examined using Western blot.

Protein degradation experiments were applied with MG132, a proteasome inhibitor. In short, after transfection for 48 h, the AGS and SGC-7901 cells transfected with pLVX-Puro-TRIM50 were evenly split into 2 groups, one group was added with 10 *μ*M of MG132, while the other one and the cells transfected with vector were added with an equal amount of dimethyl sulfoxide (DMSO). After 11 h, the cells were harvested, lysed, and *β*-catenin protein expression was analyzed using Western blot.

### 2.11. Animal Experiments

Twenty BALB/c nude mice (5-week-old, 14–20 g) supplied by Beijing Vital River Laboratory Animal Technology Co., Ltd. (Beijing, China) were used in this study. For the *in vivo* experiment, SGC-7901 cells transfected with pLVX-Puro-TRIM50 or vector were subcutaneously implanted to the mice. Then, based on the injected cells, the mice were separated into 2 groups (Vector, cells transfected with vector; TRIM50, cells transfected with pLVX-Puro-TRIM50, *n* = 6). All animals were housed in a standard environment. Tumor volume was recorded at an interval of 3 days by the formula of 0.5 × width^2^ × length from the 7th day. Thirty-one days after injection of transfected cells, the animals were euthanized, and tumors were resected, weighed, and subjected to Western blot for the detection of TRIM50, *β*-catenin, Cyclin D1, c-Myc and E-cadherin protein expression. All animal experiments were performed with the approval of the Animal Experimentation Ethics Committee of Zhejiang Eyong Pharmaceutical Research and Development Center (Certiﬁcate No. SYXK (Zhe) 2021–0033), and the experiments were conducted strictly following the guidelines of the Institutional Animal Care and Use Committee.

### 2.12. Statistical Analysis

The data of the study were presented as mean ± SD, and analyzed by SPSS 16.0. One-way ANOVA and Tukey tests were applied for multi-group comparison, and two-tailedStudent's-test was utilized for intergroup comparisons. Kruskal–Wallis H test was applied, if variances were not equal. *p* < 0.05 was considered a statistically significant difference.

## 3. Results

### 3.1. TRIM50 was Expressed at a Low Level in Human GC Tissues and Cells

**Figure 1:**
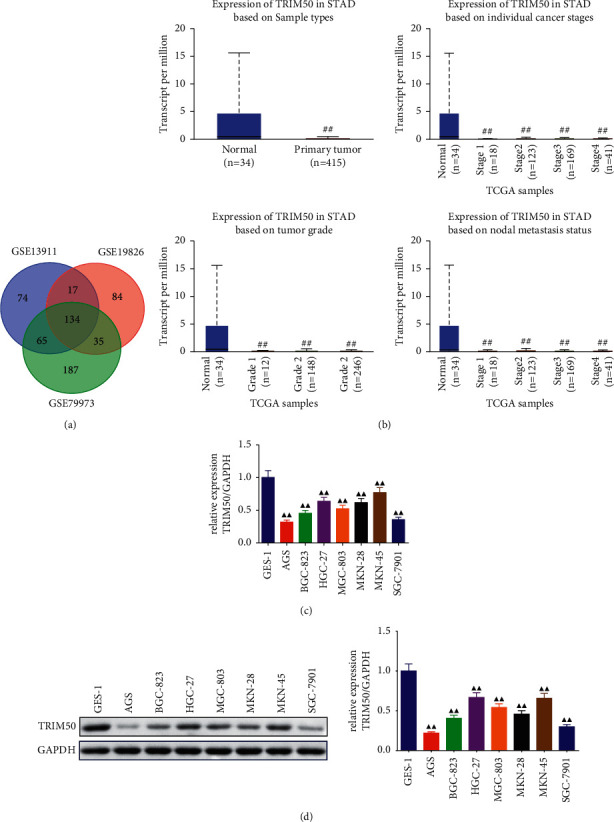
TRIM50 was expressed at a low level in GC. (a) Venn diagram showed the common genes related to GC among three datasets. (b) TCGA database revealed the relationship between TRIM50 expression and GC development. There were 34 normal samples and 415 primary GC tumor samples used for analysis. TRIM50 mRNA and protein expression of human gastric mucosal epithelial cells and GC cells were evaluated by qPCR (c) and Western blot (d). One-way ANOVA and Tukey tests were applied for multi-group comparison, and two-tailed Student's-test was utilized for intergroup comparisons. ^#^*p* < 0.05 and ^##^*p* < 0.01*vs.* Normal. ^▲^*p* < 0.05 and ^▲▲^*p* < 0.01*vs.* GES-1 group. Results were presented as mean ± SD. *n* = 3.

With the screening criterion of *p*-values <0.05 and |log_2_FC| > 1, there was 290 DEGs in the dataset of GSE1391, 270 DEGs in GSE19826, and 421 DEGs in GSE79973. As a result, 134 common DEGs related to GC were identified by the Venn diagram from GSE13911, GSE19826, and GSE79973 (the detailed information was shown in Supplemental file 1), and TRIM50 was one of the down-regulated genes among the overlapping genes (Figure 1(a)). In addition, as shown in Figure 1(b), the data revealed that TRIM50 expression was obviously lower in GC samples than in normal samples. Furthermore, relative to the normal group, the TRIM50 level was decreased in the GC patients in Stage I–IV, Grade 1–3, N0–N3. Consistently, the results of qPCR and Western blot revealed that the expression level of TRIM50 mRNA and protein was dramatically blunted in GC cells (*p* < 0.01, Figure 1(c)-1(d)). Among these GC cells, MKN-45 and HGC-27 cells exhibited higher TRIM50 expression, while AGS and SGC-7901 cells presented lower TRIM50 expression; thus, MKN-45, HGC-27, AGS, and SGC-7901 cells were selected for subsequent experiments.

### 3.2. Construction of TRIM50 Silencing and Overexpressing GC Cells

**Figure 2:**
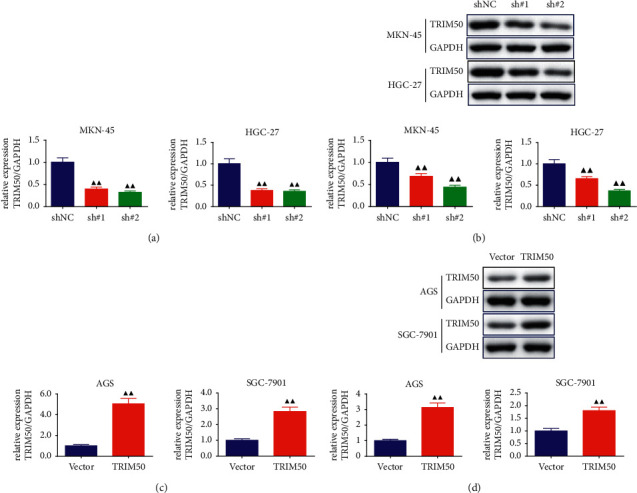
Construction and validation of TRIM50 shRNA and overexpressing GC cells. TRIM50 mRNA and protein expression in GC cells transfected with shTRIM50 (a, b) or pLVX-Puro-TRIM50 (c, d) was measured with Western blot and qPCR after 48 h of transfection. One-way ANOVA and Tukey tests were applied for multi-group comparison, and two-tailed Student's-test was utilized for intergroup comparisons. ^▲^*p* < 0.05 and ^▲▲^*p* < 0.01*vs.* shNC or Vector. Results were presented as mean ± SD. *n* = 3.

After transfection, we performed qPCR and Western blot to check the transfection efficiency. As displayed in Figure 2(a)-2(b), MKN-45 and HGC-27 cells were transfected with two separate shRNAs targeting human TRIM50 or siNC. TRIM50 knockdown successfully down-regulated TRIM50 expression at both mRNA and protein levels (*p* < 0.01). We selected sh#2 for the follow-up experiments, which exhibited relatively high inhibition efficacy on TRIM50 expression (sh#2 achieved about 70% knockdown for TRIM50 in MKN-45 cells, about 65% knockdown for TRIM50 in HGC-27 cells). In addition, as observed in Figure 2(c)-2(d), after being transfected with TRIM50 overexpressing plasmid, the expression of TRIM50 mRNA and protein was successfully increased in AGS and SGC-7901 cells (*p* < 0.01).

### 3.3. TRIM50 Inhibited the Cell Proliferation and Blocked the Cell Cycle of GC Cells

**Figure 3:**
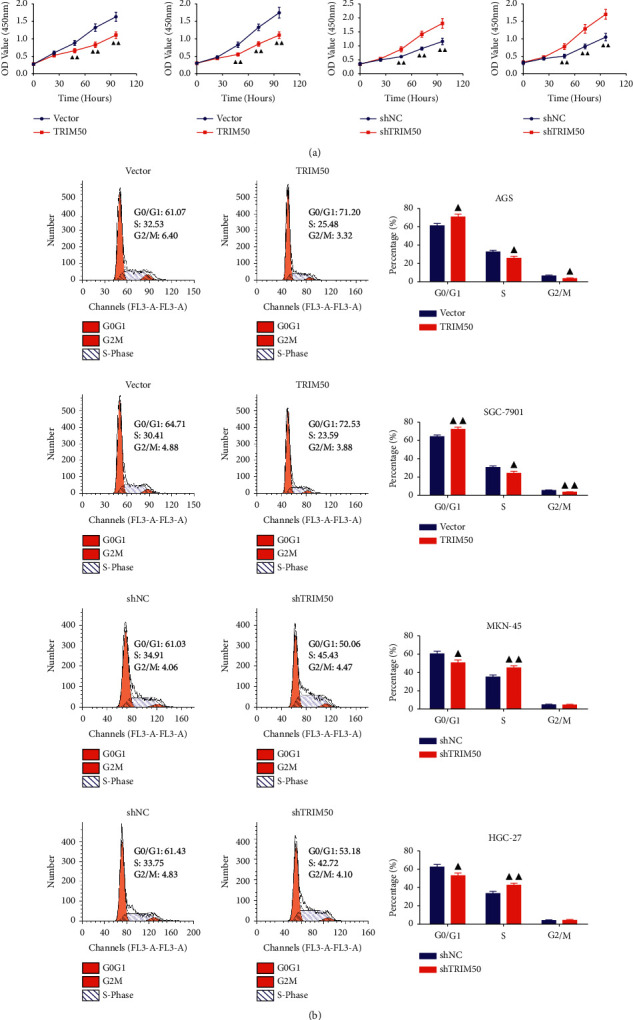
TRIM50 overexpression inhibited GC cell proliferation and blocked the cell cycle. (a) After 48 h of transfection, cell proliferation in GC cells transfected with pLVX-Puro-TRIM50 or shTRIM50 was ascertained at 0, 24, 48, 72, and 96 h with CCK-8 assay. (b) Cell cycle progression in GC cells transfected with pLVX-Puro-TRIM50 or shTRIM50 detected by flow cytometry assay after 48 h of transfection. Two-tailed Student's-test was utilized for intergroup comparisons. ^▲^*p* < 0.05 and ^▲▲^*p* < 0.01*vs.* Vector or shNC. Results were presented as mean ± SD. *n* = 3.

**Figure 4:**
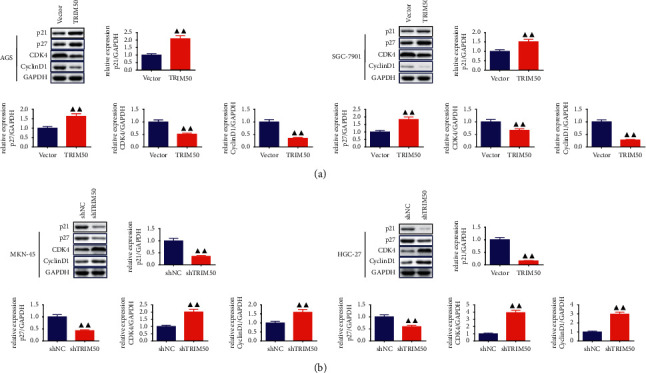
TRIM50 affected the expression of the cell cycle-related proteins in GC cells. The expression of p21, p27, CDK4, and Cyclin D1 proteins in GC cells transfected with pLVX-Puro-TRIM50 (a) or shTRIM50 (b) was detected by Western blot after 48 h of transfection. Two-tailed Student's-test was utilized for intergroup comparisons. ^▲^*p* < 0.05 and ^▲▲^*p* < 0.01*vs.* Vector or shNC. Results were presented as mean ± SD. *n* = 3.

As exhibited in Figure 3(a), we observed that TRIM50 overexpression evidently suppressed GC cells proliferation at 48, 72, and 96 h (*p* < 0.01), but TRIM50 knockdown remarkably promoted GC cells proliferation (*p* < 0.01). Moreover, TRIM50 overexpression arrested GC cells at the G0/G1 phase, while TRIM50 knockdown presented an opposite trend (*p* < 0.01, Figure 3(b)). In addition, TRIM50 overexpression could significantly elevate the protein expression of p21 and p27, and induce a reduction of CDK4 and Cyclin D1 protein expression in GC cells (*p* < 0.01). Similarly, TRIM50 knockdown had the contrary effect on the expression of these proteins in GC cells (*p* < 0.01, Figure 4).

### 3.4. TRIM50 Inhibited the Migration and Invasion of GC Cells

**Figure 5:**
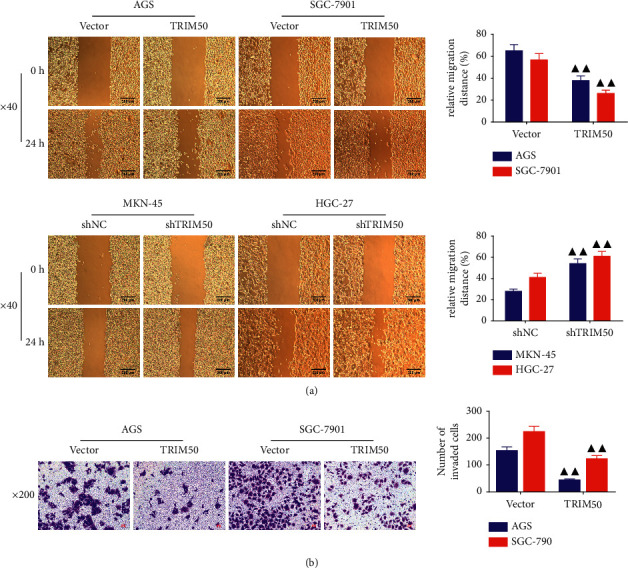
TRIM50 overexpression suppressed the migration and invasion of the GC cells. The migratory and invasive capacity of GC cells transfected with pLVX-Puro-TRIM50 or shTRIM50 was detected by scratch (a) and Transwell assay (b) after 48 h of transfection. Two-tailed Student's-test was utilized for intergroup comparisons. ^▲^*p* < 0.05 and ^▲▲^*p* < 0.01*vs.* Vector or shNC. Results were presented as mean ± SD. *n* = 3.

**Figure 6:**
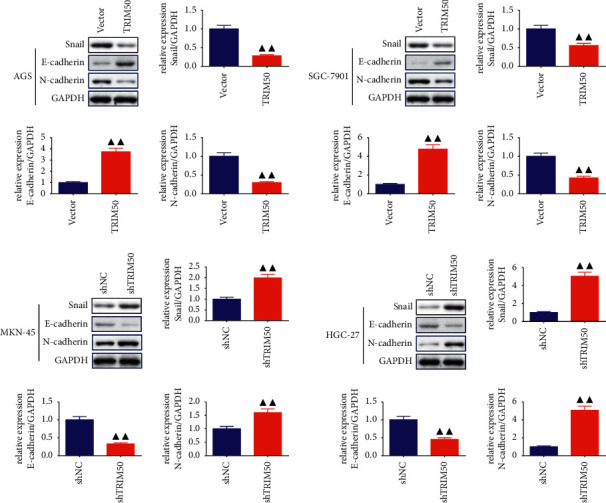
TRIM50 influenced the expression of migration- and invasion-related proteins in GC cells. The expression of Snail, E-cadherin, and N-cadherin proteins in GC cells transfected with pLVX-Puro-TRIM50 or shTRIM50 was detected by Western blot after 48 h of transfection. Two-tailed Student's-test was utilized for intergroup comparisons. ^▲^*p* < 0.05 and ^▲▲^*p* < 0.01*vs.* Vector or shNC. Results were presented as mean ± SD. *n* = 3.

In the next step, we evaluated the capacity of TRIM50 to regulate the migration and invasion of GC cells. From Figure 5(a), we observed that relative to GC cells transfected with vector, there was a notable decline in the migration ability of the GC cells transfected with pLVX-Puro-TRIM50, while TRIM50 knockdown evidently upregulated the migration ability of the GC cells (*p* < 0.01). In addition, the Transwell assay revealed that the number of invaded cells was remarkably decreased in GC cells transfected with pLVX-Puro-TRIM50 (*p* < 0.01, Figure 5(b)). To further examine whether TRIM50 was essential for modulating migration and invasion in GC cells, the expression of migration- and invasion-related proteins was assessed. As expected, we found that the expression of Snail as well as N-cadherin proteins was sharply blunted, whereas the expression of E-cadherin protein was remarkably enhanced in TRIM50 overexpressed GC cells. The opposite results were observed in TRIM50-knockdown GC cells (*p* < 0.01, Figure 6).

### 3.5. TRIM50 Interacted with *β*-Catenin to Induce Degradation of *β*-Catenin

**Figure 7:**
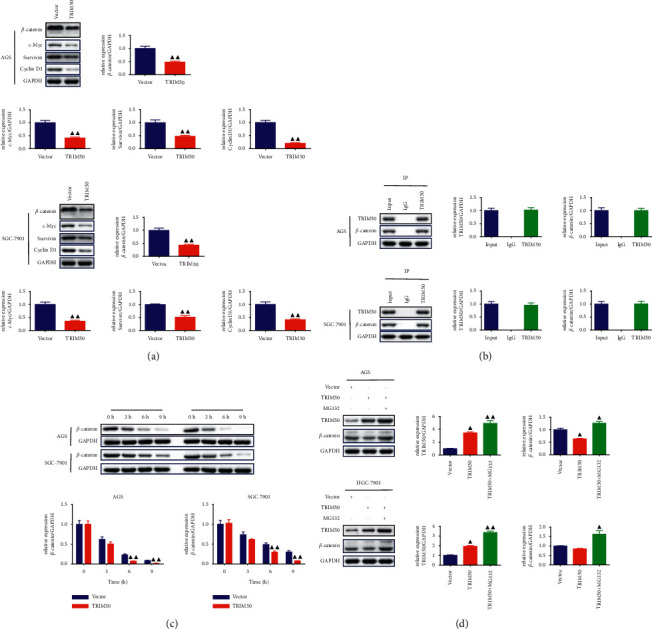
TRIM50 could interact with *β*-catenin, induce *β*-catenin degradation. (a) After 48 h of transfection, the levels of the *β*-catenin, c-Myc, survivin, and Cyclin D1 proteins in GC cells were quantified using Western blot. (b) The interaction between TRIM50 and *β*-catenin was tested with Co-IP analysis. (c, d) The effect of TRIM50 overexpression on *β*-catenin degradation was tested with the treatment of CHX (200 *μ*g/ml) and MG132 (10 *μ*M). One-way ANOVA and Tukey tests were applied for multi-group comparison, and two-tailed Student's-test was utilized for intergroup comparisons. ^▲^*p* < 0.05 and ^▲▲^*p* < 0.01*vs.* Vector or shNC. Results were presented as mean ± SD. *n* = 3.

To investigate the possible mechanisms of TRIM50 on GC, we tested the expression of *β*-catenin, c-Myc, survivin, and Cyclin D1 proteins. Analysis of Western blot indicated that there was a sharp decline in the expression of *β*-catenin, c-Myc, survivin, and Cyclin D1 proteins in TRIM50 overexpression GC cells, which hinted that TRIM50 might have an inhibitory effect on the Wnt/*β*-catenin pathway (*p* < 0.05, Figure 7(a)). To further validate the association between TRIM50 and *β*-catenin in GC, we carried out Co-IP analysis with TRIM50 and *β*-catenin antibodies. The results presented in Figure 7(b) revealed that TRIM50 efficiently interacted with *β*-catenin in GC cells. Next, we evaluated whether TRIM50 would affect the degradation of *β*-catenin with the help of CHX as well as MG132. As exhibited in Figure 7(c)-7(d), we observed that TRIM50 overexpression promoted the degradation of *β*-catenin, especially in 6 and 9 h, and TRIM50 overexpression-induced*β*-catenin degradation was effectively rescued by using MG132 (*p* < 0.05).

### 3.6. TRIM50 Inhibited GC Tumor Growth and Regulated Wnt/*β*-Catenin Signaling Pathway-Related Protein Expression in Tumor-Bearing Mice

**Figure 8:**
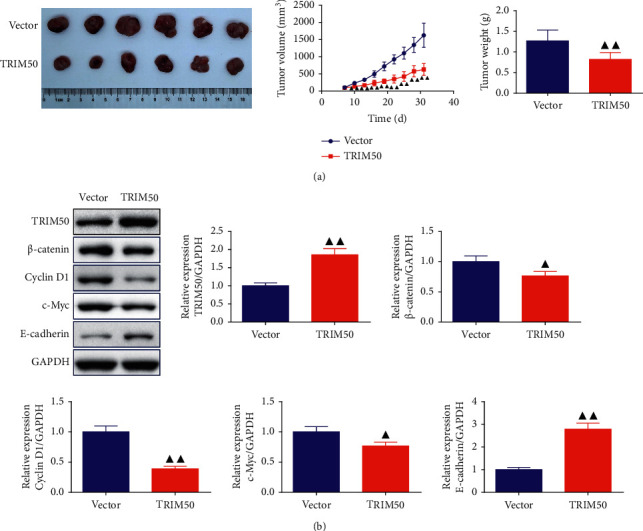
TRIM50 overexpression impeded tumor growth by influencing the Wnt/*β*-catenin pathway *in vivo*. After SGC-7901 cells were transfected with pLVX-Puro-TRIM50 or vector, the cells were subcutaneously implanted to the nude mice to set up a xenograft. (a) Tumor volume was recorded at an interval of 3 days from the 7th day and the tumor weight was valued after 31 days of injection. (b) TRIM50, *β*-catenin, Cyclin D1, c-Myc, and E-cadherin expression in the tumor tissues was detected by Western blot after 31 days of injection. Two-tailed Student's-test was utilized for intergroup comparisons. ^▲^*p* < 0.05 and ^▲▲^*p* < 0.01*vs.* Vector. Results were presented as mean ± SD. *n* = 6.

Based on the aforementioned *in vitro* findings, we performed subcutaneous xenograft experiments in nude mice to further verify the role of TRIM50 in GC development. We observed that the volume and weight of tumors formed by TRIM50 overexpression of SGC-7901 cells were markedly decreased (Figure 8(a)). Western blot showed that TRIM50 and E-cadherin were highly expressed, and *β*-catenin, Cyclin D1 as well as c-Myc were lowly expressed in TRIM50 overexpression samples (*p* < 0.05, Figure 8(b)).

### 3.7. shTRIM50 Facilitated GC Cell Proliferation and Metastasis by Promoting Wnt/*β*-Catenin Signaling Pathway *In Vitro*

**Figure 9:**
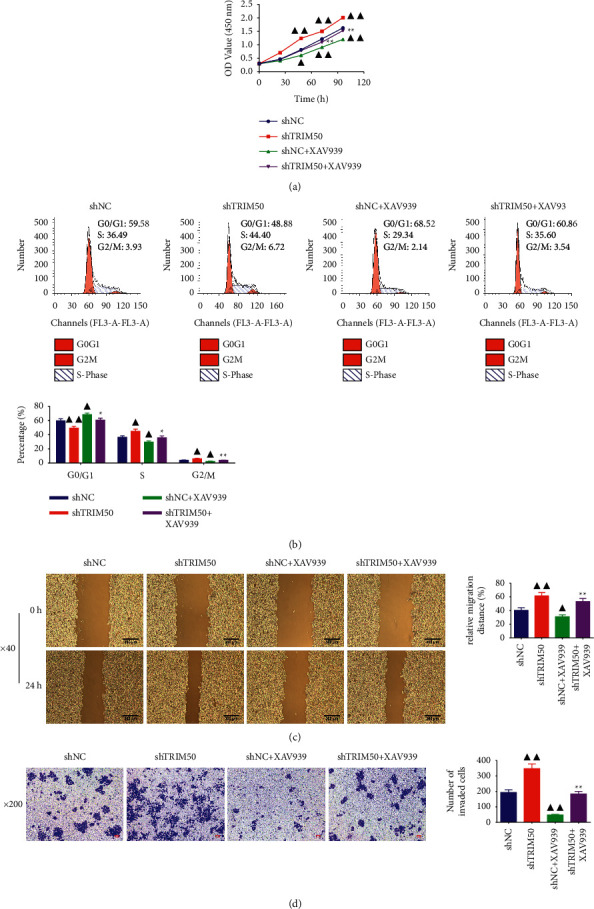
XAV939 reversed TRIM50 knockdown-promoted GC cell proliferation and metastasis. Cell proliferation, cycle progression, migration, and invasion in transfected GC cells treated with or without 10 *µ*mol/l of XAV939 were assessed with CCK-8 (a), flow cytometry assay (b), scratch assay (c), and Transwell assay (d) after 48 h of transfection. One-way ANOVA and Tukey tests were applied for multi-group comparison. ^▲^*p* < 0.05 and ^▲▲^*p* < 0.01*vs.* Vector or shNC. ^*∗*^*p* < 0.05 and ^∗∗^*p* < 0.01*vs.* shNC + XAV939. Results were presented as mean ± SD. *n* = 3.

**Figure 10:**
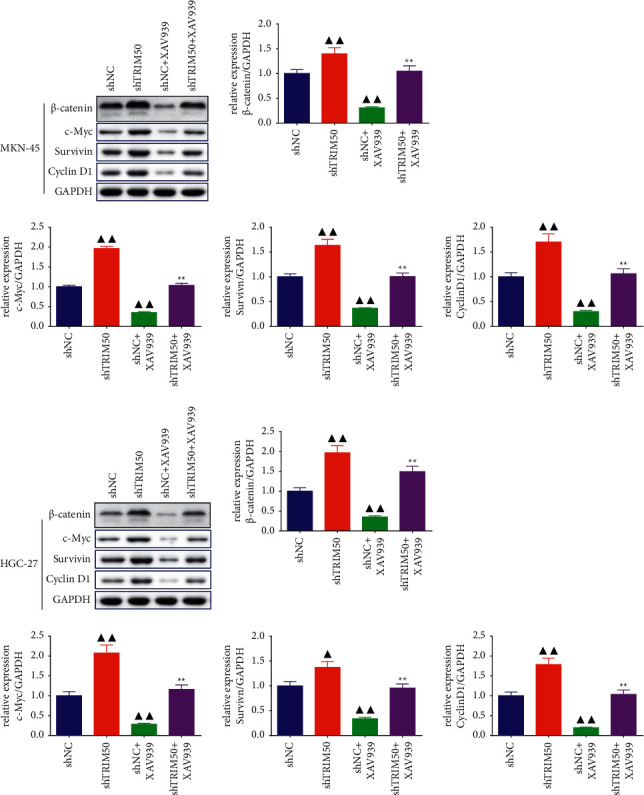
XAV939 blocked TRIM50 knockdown-induced upregulation of the Wnt/*β*-catenin pathway-related protein expression. The expression of *β*-catenin, c-Myc, survivin, and Cyclin D1 proteins in transfected GC cells treated with or without 10 *µ*mol/l of XAV939 was detected by Western blot after 48 h of transfection. One-way ANOVA and Tukey tests were applied for multi-group comparison. ^▲^*p* < 0.05 and ^▲▲^*p* < 0.01*vs.* shNC. ^*∗*^*p* < 0.05 and ^∗∗^*p* < 0.01*vs.* shNC + XAV939. Results were presented as mean ± SD. *n* = 3.

For validating the involvement of the Wnt/*β*-catenin pathway in TRIM50 knockdown-induced cell proliferation in GC, XAV939, an inhibitor of the Wnt/*β*-catenin signaling pathway, was utilized to treat MKN-45 cells transfected with shTRIM50. It was found that XAV939 not only inhibited the TRIM50 knockdown-mediated activation in cell proliferation, acceleration in cell cycle progression, but also suppressed the migratory and invasive capacity of TRIM50 knockdown GC cells (*p* < 0.01, Figure 9). Besides, as presented in Figure 10, Western blot demonstrated that TRIM50 knockdown-induced change of Wnt/*β*-cateninpathway-related proteins in GC cells was obviously rescued by the treatment of XAV939 (*p* < 0.01).

## 4. Discussion

GC has a high mortality due to its unapparent early symptoms, which has posed a tremendous health burden worldwide [19]. TRIM50 is a novel addition to the TRIM family [15]. The specific function of TRIM50 in GC oncogenesis and development is largely unclear, but more and more evidence has identified the roles of other TRIMs in GC. Among these, TRIM59 expression was enhanced in GC, which was strongly correlated with GC development and prognosis of GC patients by promoting the degradation and ubiquitination of P53 [17]. In addition, research has demonstrated that the TRIM28 gene is highly expressed in GC cells and tissues, and the intensity as well as proportion of TRIM44 positive cells could be prognosis indicators for GC patients [18]. Since all TRIM members display a high homology, they may exhibit similar functions, we hypothesize that TRIM50 might be associated with the oncogenesis and development of GC [20]. The present study using bioinformatics demonstrated that TRIM50 was down-regulated in GC samples, and TRIM50 expression was decreased with the progression of GC. Furthermore, *in vitro* assay further verified that TRIM50 was expressed at a low level in GC cells. These results were similar to previous research [15], suggesting that low TRIM50 expression may associate with poor prognosis of GC.

The etiology of GC remains not completely understood; however, similar to the majority of cancers, the progression of GC is caused by aberrant cell activities, for example, cell proliferation, cycle progression, migration, and invasion [21, 22]. Published studies have revealed that active agents targeting the proliferation, migration, and invasion of GC cells are effective strategies for GC treatment [23, 24]. It was found that overexpression of TRIM50 restrained the proliferation, invasion, as well as colony formation abilities of HCC cells, and these activities were successfully inverted by silencing the TRIM50 gene in HCC cells [15]. Consistently, in this study, we observed that TRIM50 overexpression could inhibit GC cell proliferation, cycle progression, migration as well as invasion, while TRIM50 knockdown exhibited reverse results, which suggested that TRIM50 might take part in the development of GC.

This study also investigated the molecular mechanism of how TRIM50 overexpression hampered GC tumorigenesis. As one of the highly conserved signaling pathways during biological evolution, the Wnt/*β*-catenin signaling pathway has been proved to involve in the modulation of cell proliferation, death, and differentiation by many studies [25, 26]. After years of study, researchers have inferred that there is a direct correlation between the Wnt/*β*-catenin signaling pathway and the progression of numerous cancers, including GC [27], HCC [28], and lung cancer [29]. Based on previous reports, persistent activating the Wnt/*β*-catenin signaling pathway would contribute to the hyperproliferation of stem cells, thereby inducing the initiation of tumor [30], and inhibition of the Wnt/*β*-catenin pathway could suppress the growth and migration of tumor cells [31]. Cyclin D1 is a well-known oncogene, and aberrant Cyclin D1 expression often results in an abnormal cell cycle and then facilitates tumorigenesis [32]. C-myc, as an important transcription factor, is able to influence the cell cycle via modulating the expression of cell cycle-related genes, including Cyclin D1 and p27 [11, 33]. Apart from these, both Cyclin D1 and c-Myc are crucial downstream target genes of the Wnt/*β*-catenin signaling pathway; hence, the effect of TRIM50 on the Wnt/*β*-catenin pathway could be detected by the changes of Cyclin D1 and c-Myc [34].

The results of both *in vivo* and *in vitro* experiments suggested that *β*-catenin expression was down-regulated by TRIM50 overexpression. In addition, the role of TRIM50 overexpression on the Wnt/*β*-catenin signaling pathway was further demonstrated by the decreased expression of the c-Myc, survivin, and Cyclin D1 proteins. In addition, Co-IP as well as protein stability and degradation assays revealed the relationship between TRIM50 and *β*-catenin. A published study by Zheng et al. found that inhibiting *β*-catenin degradation can promote the development of colon cancer [35]. Moreover, suppression of the Wnt/*β*-catenin pathway in GC cells by treatment with XAV939 obviously inhibited TRIM50 knockdown-induced cell proliferation, cycle progression, migration, invasion and the expression of the Wnt/*β*-cateninpathway-related proteins. These results implicated that TRIM50 may inhibit tumor proliferation and metastasis in GC via promoting *β*-catenin degradation.

In conclusion, the present study revealed the function and mechanism of TRIM50 in GC. Specifically, TRIM50 expression was decreased in GC. TRIM50 overexpression displayed inhibitory effects on tumor proliferation and metastasis in GC via regulating *β*-catenin degradation. Our study suggested that TRIM50 could be a new biomarker for predicting GC progression as well as patient prognosis, and targeting TRIM50 could offer a novel approach for GC treatment.

## Figures and Tables

**Table 1:** qPCR primers.

Gene	Forward primer	Reverse primer
Human TRIM50	GGCCCTTAGAAGGCGCATT	GCAGGGTCCAACTTGAGAGG
Human GAPDH	GGAGCGAGATCCCTCCAAAAT	GGCTGTTGTCATACTTCTCATGG

## Data Availability

Data generated from this study were included in this article.

## Supplementary Materials

Supplementary Materials Supplemental file 1: Detailed information of the 134 DEGs.
