# Supplementary material for: TRIM50 Inhibits Proliferation and Metastasis of Gastric Cancer via Promoting β-Catenin Degradation
Source: J Oncol. 2022 Aug 22;2022:5936753. doi: 10.1155/2022/5936753 (PMC9423946; doi:10.1155/2022/5936753)
Supplement: Supplementary Materials — Supplemental file 1: Detailed information of the 134 DEGs. [file 5936753.f1.docx]

| ID | adj.P.Val | P.Value | t | B | logFC | Gene.symbol | Gene.title |
| --- | --- | --- | --- | --- | --- | --- | --- |
| 207139_at | 2.52E-16 | 2.86E-20 | -12.930424 | 35.525054 | -6.765597 | ATP4A | ATPase H+/K+ transporting alpha subunit |
| 213265_at | 1.37E-12 | 1.3E-15 | -10.255404 | 25.154805 | -5.8596485 | PGA4 | pepsinogen 4, group I (pepsinogen A) |
| 220191_at | 5.35E-08 | 8.05E-10 | -7.098751 | 12.187354 | -5.7110386 | GKN1 | gastrokine 1 |
| 206334_at | 5.8E-11 | 1.57E-13 | -9.112427 | 20.498076 | -5.6887404 | LIPF | lipase F, gastric type |
| 207981_s_at | 2.11E-14 | 5.8E-18 | -11.580691 | 30.400554 | -5.1728577 | ESRRG | estrogen related receptor gamma |
| 213456_at | 3.99E-11 | 9.19E-14 | -9.239035 | 21.018358 | -5.1512823 | SOSTDC1 | sclerostin domain containing 1 |
| 202018_s_at | 1.76E-08 | 2.13E-10 | -7.412939 | 13.477971 | -5.0423431 | LTF | lactotransferrin |
| 221095_s_at | 2.29E-12 | 2.58E-15 | -10.091215 | 24.492269 | -4.9984822 | KCNE2 | potassium voltage-gated channel subfamily E regulatory subunit 2 |
| 238222_at | 3.8E-11 | 8.58E-14 | -9.255231 | 21.084851 | -4.9344177 | GKN2 | gastrokine 2 |
| 1555236_a_at | 5.89E-08 | 9.06E-10 | -7.070538 | 12.071825 | -4.8220248 | PGC | progastricsin |
| 205319_at | 2.21E-09 | 1.65E-11 | -8.016098 | 15.969157 | -4.6239056 | PSCA | prostate stem cell antigen |
| 205979_at | 8.26E-10 | 4.83E-12 | -8.304635 | 17.163225 | -4.6194744 | SCGB2A1 | secretoglobin family 2A member 1 |
| 204697_s_at | 1.84E-12 | 1.96E-15 | -10.157236 | 24.758974 | -4.592481 | CHGA | chromogranin A |
| 214046_at | 1.77E-12 | 1.8E-15 | -10.177937 | 24.842519 | -4.5341409 | FUT9 | fucosyltransferase 9 |
| 241436_at | 2.85E-11 | 5.84E-14 | -9.346637 | 21.459818 | -4.5021309 | SCNN1G | sodium channel epithelial 1 gamma subunit |
| 229177_at | 9.89E-13 | 8.32E-16 | -10.363314 | 25.588874 | -4.4897851 | C16orf89 | chromosome 16 open reading frame 89 |
| 220723_s_at | 4.24E-14 | 1.55E-17 | -11.336829 | 29.450141 | -4.475947 | CWH43 | cell wall biogenesis 43 C-terminal homolog |
| 228004_at | 2.96E-10 | 1.33E-12 | -8.608889 | 18.421567 | -4.2791925 | LINC00261 | long intergenic non-protein coding RNA 261 |
| 210381_s_at | 3.15E-13 | 1.61E-16 | -10.761191 | 27.179333 | -4.195877 | CCKBR | cholecystokinin B receptor |
| 205751_at | 1.2E-10 | 4.2E-13 | -8.879848 | 19.540214 | -3.9989636 | SH3GL2 | SH3 domain containing GRB2 like 2, endophilin A1 |
| 236430_at | 6.36E-08 | 0.000000001 | -7.047005 | 11.975515 | -3.9223444 | TMED6 | transmembrane p24 trafficking protein 6 |
| 228262_at | 2.29E-12 | 2.6E-15 | -10.088743 | 24.482277 | -3.9188629 | MAP7D2 | MAP7 domain containing 2 |
| 226960_at | 9.71E-08 | 1.71E-09 | -6.919091 | 11.452905 | -3.8068458 | CXCL17 | C-X-C motif chemokine ligand 17 |
| 228232_s_at | 2.26E-11 | 4.47E-14 | -9.409917 | 21.719105 | -3.7847629 | VSIG2 | V-set and immunoglobulin domain containing 2 |
| 1554970_at | 2.76E-09 | 2.19E-11 | -7.949654 | 15.69425 | -3.7354753 | PDILT | protein disulfide isomerase like, testis expressed |
| 206844_at | 3.21E-13 | 1.76E-16 | -10.740129 | 27.09555 | -3.7201108 | FBP2 | fructose-bisphosphatase 2 |
| 227194_at | 0.00000131 | 4.06E-08 | -6.157128 | 8.38147 | -3.6531663 | FAM3B | family with sequence similarity 3 member B |
| 203924_at | 0.00000168 | 5.47E-08 | -6.084183 | 8.092232 | -3.6320027 | GSTA1 | glutathione S-transferase alpha 1 |
| 221008_s_at | 5.39E-09 | 5.01E-11 | -7.754553 | 14.88751 | -3.556451 | ETNPPL | ethanolamine-phosphate phospho-lyase |
| 209462_at | 1.17E-10 | 3.96E-13 | -8.893615 | 19.596981 | -3.5176495 | APLP1 | amyloid beta precursor like protein 1 |
| 213953_at | 0.000000483 | 0.000000012 | -6.452049 | 9.560377 | -3.5125684 | KRT20 | keratin 20 |
| 210143_at | 1.88E-09 | 1.32E-11 | -8.068708 | 16.186858 | -3.4940284 | ANXA10 | annexin A10 |
| 243764_at | 1.09E-08 | 1.18E-10 | -7.552034 | 14.051282 | -3.4817378 | VSIG1 | V-set and immunoglobulin domain containing 1 |
| 1554182_at | 8.39E-12 | 1.24E-14 | -9.714318 | 22.962573 | -3.4383178 | TRIM74///TRIM73 | tripartite motif containing 74///tripartite motif containing 73 |
| 205464_at | 6.3E-12 | 8.76E-15 | -9.797917 | 23.302876 | -3.4206012 | SCNN1B | sodium channel epithelial 1 beta subunit |
| 238663_x_at | 2.49E-08 | 3.26E-10 | -7.312822 | 13.065983 | -3.3768752 | GRIA4 | glutamate ionotropic receptor AMPA type subunit 4 |
| 221132_at | 0.0000657 | 0.00000432 | -4.98246 | 3.877278 | -3.3721226 | CLDN18 | claudin 18 |
| 206561_s_at | 1.55E-09 | 1.02E-11 | -8.127918 | 16.431893 | -3.3373067 | AKR1B10 | aldo-keto reductase family 1 member B10 |
| 1552673_at | 2.74E-09 | 2.17E-11 | -7.951922 | 15.703635 | -3.3309151 | RFX6 | regulatory factor X6 |
| 204469_at | 2.53E-08 | 3.33E-10 | -7.30749 | 13.044057 | -3.3213445 | PTPRZ1 | protein tyrosine phosphatase, receptor type Z1 |
| 242998_at | 1E-12 | 8.63E-16 | -10.354489 | 25.553417 | -3.2654863 | RDH12 | retinol dehydrogenase 12 (all-trans/9-cis/11-cis) |
| 206212_at | 2.66E-11 | 5.41E-14 | -9.36474 | 21.534023 | -3.2610996 | CPA2 | carboxypeptidase A2 |
| 208063_s_at | 5.98E-11 | 1.64E-13 | -9.10199 | 20.455147 | -3.2252418 | CAPN9 | calpain 9 |
| 242271_at | 1.42E-10 | 5.16E-13 | -8.831292 | 19.339942 | -3.2245543 | SLC26A9 | solute carrier family 26 member 9 |
| 226281_at | 1.64E-09 | 1.1E-11 | -8.110805 | 16.361068 | -3.2157399 | DNER | delta/notch like EGF repeat containing |
| 205623_at | 9.02E-09 | 9.35E-11 | -7.607623 | 14.280659 | -3.1712082 | ALDH3A1 | aldehyde dehydrogenase 3 family member A1 |
| 223862_at | 4.51E-13 | 2.81E-16 | -10.626746 | 26.643724 | -3.1684935 | GHRL | ghrelin/obestatin prepropeptide |
| 236360_at | 5.26E-11 | 1.41E-13 | -9.138476 | 20.605193 | -3.1653568 | LINC00982 | long intergenic non-protein coding RNA 982 |
| 238862_at | 5.05E-09 | 4.62E-11 | -7.773575 | 14.966125 | -3.1632181 | MFSD4A | major facilitator superfamily domain containing 4A |
| 241137_at | 1.55E-08 | 1.8E-10 | -7.452421 | 13.640604 | -3.154979 | DPCR1 | diffuse panbronchiolitis critical region 1 |
| 1560850_at | 2.17E-08 | 2.76E-10 | -7.351806 | 13.226333 | -3.1458144 | LOC101926959 | uncharacterized LOC101926959 |
| 204777_s_at | 2.52E-16 | 3.22E-20 | -12.89971 | 35.41111 | -3.1297428 | MAL | mal, T-cell differentiation protein |
| 204810_s_at | 1.4E-09 | 8.91E-12 | -8.160802 | 16.567982 | -3.1291033 | CKM | creatine kinase, M-type |
| 206293_at | 0.0000112 | 0.000000517 | -5.527178 | 5.920993 | -3.1232566 | SULT2A1 | sulfotransferase family 2A member 1 |
| 214476_at | 0.00000251 | 0.000000088 | -5.967757 | 7.632731 | -3.1188893 | TFF2 | trefoil factor 2 |
| 238197_at | 6.72E-08 | 1.08E-09 | -7.029034 | 11.901998 | -3.0518801 | GATA5 | GATA binding protein 5 |
| 219727_at | 0.00000222 | 7.57E-08 | -6.004563 | 7.777701 | -3.0296553 | DUOX2 | dual oxidase 2 |
| 235591_at | 7.01E-08 | 1.14E-09 | -7.0159 | 11.848288 | -2.9938575 | SSTR1 | somatostatin receptor 1 |
| 207502_at | 0.000000199 | 4.1E-09 | -6.710713 | 10.605185 | -2.957866 | GUCA2B | guanylate cyclase activator 2B |
| 204704_s_at | 0.000386 | 0.0000373 | -4.402594 | 1.814924 | -2.9574457 | ALDOB | aldolase, fructose-bisphosphate B |
| 229831_at | 4.99E-08 | 7.44E-10 | -7.117188 | 12.262886 | -2.8780095 | CNTN3 | contactin 3 |
| 226582_at | 3.87E-09 | 3.25E-11 | -7.856547 | 15.309148 | -2.8563261 | LOC400043 | uncharacterized LOC400043 |
| 205041_s_at | 0.000152 | 0.0000119 | -4.713333 | 2.903819 | -2.8360112 | ORM2 | orosomucoid 2 |
| 225575_at | 2.43E-09 | 1.87E-11 | -7.986957 | 15.848582 | -2.8155074 | LIFR | leukemia inhibitory factor receptor alpha |
| 209752_at | 0.0000763 | 0.00000518 | -4.93465 | 3.702433 | -2.7932819 | REG1A | regenerating family member 1 alpha |
| 1565666_s_at | 0.0000108 | 0.000000498 | -5.536445 | 5.956508 | -2.7907433 | MUC6 | mucin 6, oligomeric mucus/gel-forming |
| 227306_at | 9.16E-12 | 1.39E-14 | -9.687013 | 22.851307 | -2.7783235 | ACER2 | alkaline ceramidase 2 |
| 222102_at | 0.000000128 | 2.41E-09 | -6.837383 | 11.119929 | -2.7719828 | GSTA3 | glutathione S-transferase alpha 3 |
| 217546_at | 1.47E-09 | 9.55E-12 | -8.144435 | 16.500248 | -2.7511341 | MT1M | metallothionein 1M |
| 226248_s_at | 0.0000137 | 0.000000656 | -5.467245 | 5.691861 | -2.7491258 | KIAA1324 | KIAA1324 |
| 210068_s_at | 0.000000355 | 8.22E-09 | -6.543749 | 9.929725 | -2.7053459 | AQP4 | aquaporin 4 |
| 207356_at | 0.00000106 | 3.12E-08 | -6.221183 | 8.636269 | -2.7047892 | DEFB4B | defensin beta 4B |
| 229070_at | 5.56E-10 | 2.97E-12 | -8.419042 | 17.636576 | -2.6782117 | ADTRP | androgen dependent TFPI regulating protein |
| 1553797_a_at | 1.72E-08 | 2.07E-10 | -7.420086 | 13.507405 | -2.6724931 | LINC01105 | long intergenic non-protein coding RNA 1105 |
| 205295_at | 9.72E-11 | 3.15E-13 | -8.947937 | 19.820891 | -2.6714973 | CKMT2 | creatine kinase, mitochondrial 2 |
| 1552834_at | 1.81E-09 | 1.25E-11 | -8.080626 | 16.236177 | -2.6661979 | B3GNT6 | UDP-GlcNAc:betaGal beta-1,3-N-acetylglucosaminyltransferase 6 |
| 209301_at | 5.08E-08 | 7.62E-10 | -7.11175 | 12.240608 | -2.6579256 | CA2 | carbonic anhydrase 2 |
| 217187_at | 0.000000941 | 2.69E-08 | -6.256949 | 8.778852 | -2.6343745 | MUC5AC | mucin 5AC, oligomeric mucus/gel-forming |
| 210505_at | 4.61E-13 | 3.12E-16 | -10.600983 | 26.54087 | -2.631883 | ADH7 | alcohol dehydrogenase 7 (class IV), mu or sigma polypeptide |
| 215129_at | 1.09E-08 | 1.19E-10 | -7.551674 | 14.049795 | -2.6019888 | PIK3C2G | phosphatidylinositol-4-phosphate 3-kinase catalytic subunit type 2 gamma |
| 1564333_a_at | 1.62E-15 | 3.56E-19 | -12.283207 | 33.097315 | -2.5903015 | PSAPL1 | prosaposin-like 1 (gene/pseudogene) |
| 238018_at | 1.6E-09 | 1.06E-11 | -8.119907 | 16.398737 | -2.5854877 | FAM150B | family with sequence similarity 150 member B |
| 225491_at | 2.54E-08 | 3.34E-10 | -7.306909 | 13.041668 | -2.5780812 | SLC1A2 | solute carrier family 1 member 2 |
| 215103_at | 0.000000038 | 5.4E-10 | -7.193333 | 12.575132 | -2.5314358 | CYP2C18 | cytochrome P450 family 2 subfamily C member 18 |
| 1553798_a_at | 1.44E-08 | 1.65E-10 | -7.473222 | 13.72632 | -2.4909356 | FBXL13 | F-box and leucine rich repeat protein 13 |
| 210179_at | 3.24E-08 | 4.49E-10 | -7.237055 | 12.754619 | -2.4401198 | KCNJ13 | potassium voltage-gated channel subfamily J member 13 |
| 209309_at | 0.00000042 | 1.02E-08 | -6.492229 | 9.722064 | -2.4232623 | AZGP1 | alpha-2-glycoprotein 1, zinc-binding |
| 235050_at | 0.00000201 | 6.78E-08 | -6.031844 | 7.885331 | -2.4008627 | SLC2A12 | solute carrier family 2 member 12 |
| 211470_s_at | 0.000000313 | 7.12E-09 | -6.578447 | 10.069795 | -2.400133 | SULT1C2 | sulfotransferase family 1C member 2 |
| 205650_s_at | 0.000000969 | 2.79E-08 | -6.248258 | 8.744185 | -2.3762978 | FGA | fibrinogen alpha chain |
| 229638_at | 5.67E-10 | 3.11E-12 | -8.408212 | 17.591774 | -2.3566618 | IRX3 | iroquois homeobox 3 |
| 210065_s_at | 0.0000171 | 0.000000861 | -5.398281 | 5.429421 | -2.3514655 | UPK1B | uroplakin 1B |
| 239270_at | 1.94E-08 | 2.39E-10 | -7.386176 | 13.367781 | -2.298784 | PLCXD3 | phosphatidylinositol specific phospholipase C X domain containing 3 |
| 214420_s_at | 0.000000442 | 1.08E-08 | -6.47847 | 9.66667 | -2.2909604 | CYP2C9 | cytochrome P450 family 2 subfamily C member 9 |
| 234367_x_at | 6.22E-09 | 5.95E-11 | -7.713954 | 14.719755 | -2.2754967 | TMPRSS6 | transmembrane protease, serine 6 |
| 223720_at | 6.61E-08 | 1.06E-09 | -7.033176 | 11.918941 | -2.2502765 | SPINK7 | serine peptidase inhibitor, Kazal type 7 (putative) |
| 222901_s_at | 0.000000536 | 1.36E-08 | -6.42217 | 9.440299 | -2.2360392 | KCNJ16 | potassium voltage-gated channel subfamily J member 16 |
| 236308_at | 1.64E-08 | 1.93E-10 | -7.436471 | 13.574894 | -2.2293642 | VSTM2A | V-set and transmembrane domain containing 2A |
| 228462_at | 0.00189 | 0.000274 | -3.831727 | -0.071619 | -2.2095885 | IRX2 | iroquois homeobox 2 |
| 231463_at | 0.00000256 | 0.00000009 | -5.962011 | 7.610125 | -2.2037008 | CNTD1 | cyclin N-terminal domain containing 1 |
| 206310_at | 6.45E-08 | 1.03E-09 | -7.04109 | 11.951316 | -2.1943371 | SPINK2 | serine peptidase inhibitor, Kazal type 2 |
| 210807_s_at | 1.05E-08 | 1.13E-10 | -7.561917 | 14.092055 | -2.188605 | SLC16A7 | solute carrier family 16 member 7 |
| 211806_s_at | 5.34E-09 | 4.93E-11 | -7.758235 | 14.902725 | -2.1834872 | KCNJ15 | potassium voltage-gated channel subfamily J member 15 |
| 205927_s_at | 0.000000108 | 1.94E-09 | -6.889626 | 11.332749 | -2.164674 | CTSE | cathepsin E |
| 205199_at | 6.41E-09 | 6.18E-11 | -7.705239 | 14.683748 | -2.093642 | CA9 | carbonic anhydrase 9 |
| 229909_at | 1.42E-10 | 5.18E-13 | -8.830282 | 19.335775 | -2.0868759 | B4GALNT3 | beta-1,4-N-acetyl-galactosaminyltransferase 3 |
| 205509_at | 0.00000282 | 0.000000101 | -5.934038 | 7.500164 | -2.0754952 | CPB1 | carboxypeptidase B1 |
| 225458_at | 7.19E-08 | 1.18E-09 | -7.008541 | 11.818203 | -2.071523 | PP7080 | uncharacterized LOC25845 |
| 244509_at | 6.87E-10 | 3.94E-12 | -8.352698 | 17.3621 | -2.0604373 | GPR155 | G protein-coupled receptor 155 |
| 211766_s_at | 0.00179 | 0.000257 | -3.850347 | -0.012663 | -2.0541293 | PNLIPRP2 | pancreatic lipase related protein 2 (gene/pseudogene) |
| 229019_at | 0.000000378 | 8.93E-09 | -6.523934 | 9.849815 | -2.0485244 | ZNF385B | zinc finger protein 385B |
| 207909_x_at | 0.0181 | 0.00476 | -2.915475 | -2.720874 | -2.0332704 | DAZ4 | deleted in azoospermia 4 |
| 231018_at | 0.000000623 | 1.64E-08 | -6.377429 | 9.260749 | -2.017368 | PALM3 | paralemmin 3 |
| 205009_at | 0.00000605 | 0.000000252 | -5.70745 | 6.615817 | -2.0051655 | TFF1 | trefoil factor 1 |
| 211549_s_at | 0.00000572 | 0.000000235 | -5.724562 | 6.682192 | -1.9167556 | HPGD | hydroxyprostaglandin dehydrogenase 15-(NAD) |
| 220794_at | 0.000103 | 0.00000747 | -4.838107 | 3.351838 | -1.9130806 | GREM2 | gremlin 2, DAN family BMP antagonist |
| 1563407_x_at | 2.55E-10 | 1.1E-12 | -8.653204 | 18.604682 | -1.795985 | ATP4B | ATPase H+/K+ transporting beta subunit |
| 1552911_at | 0.000158 | 0.0000125 | -4.700855 | 2.859338 | -1.7137787 | SIGLEC11 | sialic acid binding Ig like lectin 11 |
| 242913_at | 0.00000363 | 0.000000138 | -5.856934 | 7.197945 | -1.6139889 | CLIC6 | chloride intracellular channel 6 |
| 211829_s_at | 0.0000409 | 0.00000241 | -5.134062 | 4.43683 | -1.5623707 | GPER1 | G protein-coupled estrogen receptor 1 |
| 1552801_at | 0.00224 | 0.00034 | -3.76676 | -0.275858 | -0.9660489 | CAPN13 | calpain 13 |
| 208482_at | 0.00924 | 0.00201 | -3.207677 | -1.933036 | -0.904592 | SSTR1 | somatostatin receptor 1 |
| 1570015_at | 0.00528 | 0.000998 | -3.434972 | -1.281898 | -0.8765634 | CHIAP2 | chitinase, acidic pseudogene 2 |
| 1552827_s_at | 0.00107 | 0.000133 | -4.043391 | 0.609227 | -0.7942917 | SLC26A7 | solute carrier family 26 member 7 |
| 231014_at | 0.0492 | 0.0174 | -2.436687 | -3.881438 | -0.7930985 | TRIM50 | tripartite motif containing 50 |
| 235194_at | 0.000814 | 0.0000944 | -4.141924 | 0.93389 | -0.6887144 | TPCN2 | two pore segment channel 2 |
| 217175_at | 0.108 | 0.0484 | -2.009088 | -4.768246 | -0.6230646 | UGT2B15 | UDP glucuronosyltransferase family 2 member B15 |
| 239365_at | 0.0774 | 0.0314 | -2.19642 | -4.39797 | -0.52737 | SCIN | scinderin |
| 206889_at | 0.00326 | 0.000541 | -3.62578 | -0.711061 | -0.4817666 | PDIA2 | protein disulfide isomerase family A member 2 |
| 242730_at | 0.0174 | 0.00453 | -2.932789 | -2.675806 | -0.4156408 | MYRIP | myosin VIIA and Rab interacting protein |
| 217835_x_at | 0.034 | 0.0108 | 2.61844 | -3.460839 | 0.2634547 | TGIF2-C20orf24 | TGIF2-C20orf24 readthrough |
| 1554553_s_at | 0.0012 | 0.000154 | 4.001525 | 0.472736 | 0.3901118 | YIF1B | Yip1 interacting factor homolog B, membrane trafficking protein |
| 224465_s_at | 0.00281 | 0.00045 | 3.682078 | -0.538605 | 0.4789609 | PYM1 | PYM homolog 1, exon junction complex associated factor |
